# Cognition in vestibular disorders: state of the field, challenges, and priorities for the future

**DOI:** 10.3389/fneur.2024.1159174

**Published:** 2024-01-18

**Authors:** Laura J. Smith, David Wilkinson, Mayur Bodani, S. S. Surenthiran

**Affiliations:** ^1^Centre for Preventative Neurology, Wolfson Institute of Population Health, Queen Mary University of London, London, United Kingdom; ^2^School of Psychology, Keynes College, University of Kent, Kent, United Kingdom; ^3^The London Neuro-otology Centre, London, United Kingdom

**Keywords:** vestibular disorders, cognition, psychological distress, assessment practices, rehabilitation

## Abstract

Vestibular disorders are prevalent and debilitating conditions of the inner ear and brain which affect balance, coordination, and the integration of multisensory inputs. A growing body of research has linked vestibular disorders to cognitive problems, most notably attention, visuospatial perception, spatial memory, and executive function. However, the mechanistic bases of these cognitive sequelae remain poorly defined, and there is a gap between our theoretical understanding of vestibular cognitive dysfunction, and how best to identify and manage this within clinical practice. This article takes stock of these shortcomings and provides recommendations and priorities for healthcare professionals who assess and treat vestibular disorders, and for researchers developing cognitive models and rehabilitation interventions. We highlight the importance of multidisciplinary collaboration for developing and evaluating clinically relevant theoretical models of vestibular cognition, to advance research and treatment.

## 1 Introduction

Vestibular disorders are common [11.9% of adults in the United States have a dizziness or balance problem (1); 6.5% have a peripheral disorder (2)], and prevalence increases with age (3). Vestibular disorders are disabling conditions which adversely impact quality of life, psychological wellbeing, daily activities, and employment, and place strain on healthcare resources. Up to 60% of people with vestibular disorders experience cognitive problems (4). Common complaints include brain fog and trouble concentrating, difficulties multi-tasking, word-finding problems, and forgetting in everyday life (5–7). Cognitive problems are among the strongest predictors of disability for people with vestibular disorders and interfere with clinical recovery (8, 9).

The past 25 years have seen developments in our understanding of the epidemiology, expression, and neural bases of vestibular cognitive dysfunction. This understanding offers important insights into how vestibular signals contribute to cognition within both health and disease. However, key theoretical and methodological gaps need to be addressed before this understanding can inform clinical practice. This narrative review aims to summarize key vestibular-cognitive literature relevant to this Frontiers Research Topic, drawing on existing reviews, and adding contemporary evidence which has not yet been reviewed. This review will identify some important knowledge gaps and make recommendations for future work. Our intention was not to produce a comprehensive literature review, but rather a statement of what we understand to be critical research priorities.

## 2 Vestibulo-cortical network

The peripheral vestibular structures originate from the statolithic system dating back 635 million years (10, 11). The vestibular pathways that stem from this are widely distributed, reaching almost all levels of the central nervous system, including widespread projections to the cerebral cortices (12, 13). Unlike other senses, the vestibulo-cortical network is multimodal and does not include a primary sensory cortex (14, 15). The nature of this anatomical distribution, which we review below, lends some clarity to why and how the vestibular system modulates cognitive function and emotion regulation (16).

### 2.1 Vestibular-spinal pathways

The vestibulospinal pathways, projecting from the vestibular nuclei to the spinal motor neurons, consist of the lateral and medial vestibulospinal tracts, and the lateral and medial reticulospinal pathways (17). These pathways, acting mainly through vestibulospinal reflexes, are involved in posture, balance, and spatial orientation.

The lateral vestibulospinal tract arises almost entirely from the lateral vestibular nucleus, with just a small component from the dorsal vestibular nucleus. This ipsilateral pathway stretches the length of the spinal cord and is involved in limb control and axial musculature (18). The descending fibers in this tract are mostly excitatory but can exert an inhibitory influence on motor neurons via spinal inhibitory interneurons. The medial vestibulospinal tract originates in the rostral part of the descending vestibular nucleus and the adjacent areas of the medial and lateral vestibular nuclei (18). Axons in this pathway descend bilaterally in the ipsilateral and contralateral medial longitudinal fasciculus and are important in bringing the axial neck and trunk musculature under reflex control of the semicircular canals. Most of the fibers from this tract are in the cervical spinal cord, although some descend to the thoracic region (18).

Descending reticulospinal pathways originate in the punto-medullary reticular formation and provide an alternative route to the vestibulospinal tracts by which vestibular signals can reach the spinal cord (18). They are divided into the medial reticulospinal tract (originating from the pontine regions and nuclei of the brainstem tegmentum) and lateral reticulospinal tract (arises more caudally). Both these pathways are bilateral, but with an ipsilateral predominance. Both descend through the brainstem and spinal cord, and although some fibers terminate in the cervical and thoracic regions, many reach the lumbar area. The neurones of both these tracts contribute to postural control, receiving numerous presynaptic vestibular inputs. Further, these tracts, like the vestibular nuclei, receive direct input from the cerebral cortex, mainly from the motor and pre-motor cortex (19).

### 2.2 Vestibular-oculomotor pathways

Foveal stabilization of images is essential for maintaining visual acuity during head motion and is achieved by the three-neuron reflex arc of the vestibulo-ocular reflex (VOR). Namely the primary sensory afferent neuron in the vestibular nerve, the neurone in the vestibular nucleus within the ponto-medullary region, and the motor neurones within the oculomotor nuclei in the midbrain (12). The head motion signal (acceleration) of the semicircular canal is converted into an eye position signal via integration, firstly within the labyrinth, and then by the common oculomotor integrator, a network of neurones within the brainstem and deep cerebellar nuclei (20). Additionally, there is a central integrative network located mainly within the medial and superior vestibular nuclei and their commissural interconnections (21). The vestibulocerebellum has a modulating effect on the VOR (22), including cancelation (23) or suppression (24), which is essential for many aspects of normal activity. The cerebellum has an essential role in VOR motor learning (25, 26) as well as in motion perception (20).

The VOR can be cognitively suppressed by asking a subject to follow an imagined target (no visual feedback) by moving the eyes and head together (27). Moreover, the compensation of unilateral peripheral vestibular deficits is dependent upon ongoing cognitive input (28). Those with anxiety also demonstrate significantly higher VOR gain and shorter VOR time constant (25). The finding that caloric stimulation, which activates the VOR pathway, also produces activity within cortical, subcortical, and cerebellar structures (29, 30) underlines the modulatory role that cortical areas involved in cognition have on the VOR in both normal day-to-day functioning and pathological states.

### 2.3 Vestibular-cerebellar pathways

The vestibulo-cerebellum is, in phylogenetic terms, the oldest part of the cerebellum and is involved in sensorimotor integration, motor control, and providing precision to movements. The peripheral and central vestibular structures project fibers to the cerebellum via three main pathways: primary, secondary, and tertiary (13, 18).

Within the primary afferent pathway, mossy fiber afferents from each of the five parts of the labyrinth combine to form the vestibular nerve, which as it approaches the brainstem, splits into two branches. The thicker of these branches enters the medulla and ends in the medial, descending, and superior vestibular nuclei. The thinner branch enters and passes through the superior lateral vestibular nuclei, ascending and ending in the ipsilateral uvula-nodulus of the cerebellum (18). However, the length of the parallel fibers ensures that the Purkinje fibers in a single folium and receive inputs from both ipsilateral and contralateral labyrinths. Secondary mossy fiber afferents project from the caudal aspects of the medial, descending, and superior vestibular nuclei bilaterally mainly to the uvula-nodulus and flocculus of the cerebellum, but also to the anterior vermis and paraflocculus. Finally, tertiary vestibular projections from the two sub-nuclei of the inferior olive, the ß-nucleus, and the dorso-medial cell column project to the contralateral uvula-nodulus (18). As noted above, these sub-nuclei received direct projections from the parasolitary nucleus.

### 2.4 Vestibular-thalamo-cortical pathways

The thalamus plays a critical role in perception, receiving information from multiple sensory systems (including the vestibular system) and conveying both unprocessed signals and modulated sensory information to the cortex (31). Thalamic nuclei, which receive vestibular projections, also receive inputs from other sensory systems and there is evidence that these are integrated at the thalamic level (32). This multi-sensory integration likely plays a role in postural stability [thalamic integration of vestibular and proprioceptive inputs (33)], and the perception of visual vertical [thalamic integration of visual and vestibular inputs (34, 35)].

There are multiple projections from the vestibular nuclei in the brainstem to the different thalamic nuclei, including projections to the ventroposterior complex, ventroanterior and ventrolateral nuclear complex, intralaminar nuclei, and thalamic posterior nuclear group (15, 36). Onward projections are then sent to multiple vestibular areas of the cerebral cortex including the parietal cortex, parieto-insular vestibular cortex, temporo-parietal junction, frontal cortex, cingulate, and the striate/extrastriate visual cortices (13, 37–40). In turn, these areas of the vestibular cortex, send corticofugal fibers to the vestibular nuclei, the parabrachial nuclei, and the nucleus prepositus hypoglossi in the brainstem (15). Electrophysiological studies show that this corticofugal input to neurones within the vestibular nuclei is complex and can be inhibitory, facilitatory, or a combination of both. Thalamic-vestibular neurons can also distinguish between translations and head tilts in relation to gravity, and between active and passive head movements (15).

Vestibular-thalamic projections to the vestibular and entorhinal cortices provide the basis for self-motion perception and spatial orientation, respectively (41). The posterior vestibulo-thalamic pathway, consisting of the posterior lateral nucleus, projects to the parts of the cortex responsible for self-motion perception. Meanwhile, the anterior vestibulo-thalamic pathway extends from the nucleus prepositus and supragenual nucleus to the head direction network. Both play an important role in cognitive and sensorimotor functioning by providing motor control and perceptual stability in day-to-day activities (42).

### 2.5 Vestibular-hippocampal pathways

In animal models, stimulation of the medial vestibular nucleus elicits firing of the hippocampal subfield CA1 spike cells, which likely correspond to place cells in the hippocampus (43). Functional magnetic resonance imaging (fMRI) studies in healthy participants following cold caloric irrigation show activation of the hippocampus as well as activation of the retrosplenial cortex and subiculum, indicating these activations were related to the feelings of spatial disorientation and self-rotation experienced by the participants during caloric stimulation (44). Lesions of the peripheral vestibular structures alter hippocampal place cell function, electroencephalogram (EEG) activity, and CA1 field potentials in rats (45). Moreover, numerous studies with animals and humans demonstrate peripheral vestibular dysfunction leads to deficits in spatial memory and navigation (46–48).

### 2.6 Vestibular-cortical–cortical pathways

Functional imaging studies have revealed a stable network of vestibular cortical connectivity, with right hemisphere dominance and redundancy, which could explain why cortical lesions in humans do not usually result in lasting vestibular symptoms (49). Overall, there is a wide-ranging inter-connectivity between vestibular cortical areas, centered largely around the parietoinsular vestibular cortex (PIVC) (39). There are also direct cortical projections to the vestibular nuclei, prepositus hypoglossi, and parabrachial nucleus (50). The response of these areas to multimodal inputs and their interaction with each other results in spatial orientation and motion perception, the nature of which is currently poorly understood (39).

Five main areas of the cortex have been shown to respond to vestibular stimulation (51):

- Area 2v–lies at the base of the intra-parietal sulcus. Electrical stimulation of this region in humans results in sensations of whole-body motion (52).
- Area 3a, located in the base of the central sulcus next to the motor cortex, receives inputs from the ventro-postero-lateral and ventro-posterior inferior thalamic nuclei. The cells in this region project onto area 4 of motor cortex and therefore maybe involved with motor control (51).
- In area 7 of the parietal cortex, vestibular neurons have been found in the posterior parietal cortex (13). The ventral intraparietal area also contains multimodal neurons that respond to spatial coding. The medial intraparietal area and medial superior temporal area also respond to visual and vestibular motion signals and integrate signals regarding body motion through space. Pathological lesions in this region can cause confusion of spatial awareness.
- The PIVC is in the retro-insular and granular insula regions of the lateral sulcus. Many of the neurons in this area respond to visual, somatosensory, proprioceptive, and body motion stimuli (53). Those with pathological lesions in this area frequently experience vertigo, unsteadiness, and loss of visual vertical perception (39).
- Area OP2 in the parietal operculum is proposed to be the human equivalent of the non-human primate PIVC because of its specific and robust response to vestibular stimulation, its dense connections with cortical and subcortical vestibular structures (including the posterior insula), and functional responses (54, 55). OP2 receives vestibular, visual, and somatosensory inputs and is thought to process multisensory information relevant to higher order vestibular functions including perception of self-motion and verticality (55).

- Area 6 of the prefrontal gyrus and superior frontal gyrus, receives vestibular input. These areas are related to the frontal eye field, which is involved in the control of saccades and smooth pursuit (51).

## 3 Cognitive profile

The most widely reported form of vestibular-mediated cognitive dysfunction relates to spatial cognition (memory, navigation, mental rotation, and representation) (56). Slowed processing speed, disturbances to attention, short-term memory, arithmetic, and executive function are also reported, albeit less commonly (8).

Currently, we know relatively little about how cognitive profiles might vary across individuals, and there is limited evidence of how long vestibular cognitive dysfunction persists in humans (57, 58). We do however know that cognitive decline in people with vestibular disorders is more prevalent with increasing age (59), and that most vestibular disorders are more prevalent in females, with some emerging evidence that women exhibit greater spatial navigation and memory deficits (60). Vestibular symptom severity, duration, and degree of compensation can also influence cognitive impairment (61, 62).

The extent of vestibular dysfunction may also relate to the profile of cognitive impairment (63, 64). Different patterns of spatial memory and navigation impairments have been demonstrated among persons with bilateral, partial, and unilateral vestibular loss (65). In particular, navigational skill (as measured by traveled paths, heading errors, time spent in target location) and hippocampal atrophy show stronger and more consistent effects in patients with bilateral compared to unilateral vestibular loss (13, 48, 65), and also correlate with the severity of bilateral vestibulopathy (66). Moreover, patients with complete vestibular loss show different navigation-induced brain activations; patients with partial loss tend to place more reliance on neural networks involved in head direction calculations, while complete loss patients tend to recruit networks for stimulus–response learning (67). Gammeri et al. (68) also found that chronic bilateral vestibular loss decreases reliance on allocentric reference frames associated with hippocampal and entorhinal activations, while unilateral vestibular loss decreases the likelihood of using egocentric reference frames associated with the fronto-temporo-parietal network.

Some cognitive variation can also be expected across vestibular disorders since each has a distinct etiology which could differentially impact cognitive function. Below we summarize available literature exploring cognitive function in common peripheral and central vestibular disorders.

### 3.1 Peripheral disorders

Peripheral disorders affect the labyrinth and vestibular nerve. Benign paroxysmal positional vertigo (BPPV), vestibular neuritis, and Ménière’s disease are among the most frequently observed peripheral disorders. Comprehensive evidence is lacking about the cognitive problems experienced by this cohort and, more specifically, the extent to which this compromises their functional independence. Some interesting observations have nevertheless been reported.

Epidemiological studies have demonstrated that patients with loss of otolith function measured using vestibular-evoked myogenic potentials show poorer performance on spatial memory tasks, driving difficulties, and an increased risk of dementia (69, 70). After adjusting for demographic factors and medical history, older adults with BPPV are also at increased risk of dementia; a susceptibility that may stem from an increased falls risk and physical inactivity (71).

Moser et al. (72) compared the numerical cognition abilities of 20 participants with vestibular neuritis against a healthy control sample. Patients performed worse in a math’s assessment but showed normal number magnitude processing within a Stroop task. Impaired executive functions were thought to underlie this deficit (i.e., updating and replacing items in working memory to solve an arithmetic problem), rather than a specific number processing deficit (dyscalculia). The authors attributed this deficit to metabolic downregulations in inferior parietal areas associated with numerical cognition among peripheral vestibular disorders generally, rather than a specific pathology in patients with vestibular neuritis.

In other research, Candidi et al. (73) showed that patients with BPPV and vestibular neuritis both had impaired performance on mental rotation tasks (egocentric own-body and allocentric human figures) relative to healthy controls, with vestibular neuritis patients performing worse overall. The finding supports the idea that different forms of peripheral dysfunction can cause mental rotation deficits. Since BPPV and vestibular neuritis are characterized by different symptoms, the authors suggest altered central vestibular processing may underlie the rotational impairments observed.

Looking beyond BPPV and vestibular neuritis, a limited number of studies have examined cognitive function in Ménière’s disease. Gallardo-Flores (74) evaluated spatial memory in patients with different stages of unilateral and bilateral Ménière’s disease using the Virtual Morris Water Maze paradigm. Spatial memory performance (as measured by response time, accuracy and paths traveled) was reduced in the Ménière’s disease group relative to healthy controls, with patients in the latter stages of Ménière’s disease (III-IV) showing slower response times relative to those in earlier stages (I-II). Eraslan Boz et al. (75) also compared the performance of patients with unilateral and bilateral Ménière’s disease to a healthy control sample on a detailed neuropsychological battery. Attention, memory, visuospatial constructional ability, information processing, and executive function were all impaired in the Ménière’s disease sample relative to the control group. Reassuringly, Zhong et al. (76) showed that Ménière’s disease patients’ scores on the Montreal Cognitive Assessment (MoCA) improved after receipt of a stepped therapy protocol to reduce the severity of vestibular symptoms, across three follow-up timepoints (3, 6, 12 months).

### 3.2 Central disorders

Disorders of the central vestibular system involve the vestibular nuclei and the vestibular pathways that project from these nuclei to the vestibulo-cerebellum. These are often more challenging to diagnose, despite the relative prevalence of vestibular migraine (77).

Compared to migraine patients, vestibular migraine patients have more severe cognitive impairment (global, memory, executive function, verbal fluency), increased brain white matter lesions, more severe brainstem dysfunction, and lower quality of life (76, 78). In line with this neuropsychological profile, patients with vestibular migraine are more likely to self-report difficulty concentrating and remembering relative to survey respondents without dizziness (79) and those with Ménière’s disease or BPPV (61, 80). On a cautionary note, Demirhan and Celebiosy (81) failed to show any differential cognitive performance among patients with vestibular migraine and Ménière’s disease relative to a healthy control group. Patients with vestibular migraine did however self-report higher anxiety levels compared to controls.

Felfela and et al. (82) examined elderly patients with dizziness using neuro-otological tests, the MoCA, and an MRI scan. Preliminary data showed significant correlations between MoCA scores and brain atrophy in insular and temporo-parietal regions with right-sided dominance. Caloric mean excitability did not correlate with these brain regions, perhaps suggesting central multisensory vestibular processing networks, not peripheral vestibular pathology, are most associated with cognitive decline in elderly patients with cognitive impairment. Recently, Ibitoye et al. (83) also found that patients with idiopathic (unexplained) dizziness show lower frontal white matter integrity, reduced diffuse structural connectivity (encompassing central vestibular networks), impaired executive function, and balance problems (postural control, gait). The results indicate cerebral small vessel disease is involved in balance dysfunction, with idiopathic dizziness a potential predictor of balance and cognitive impairment.

A common cause of central vestibular dysfunction is a stroke or traumatic brain injury affecting the cerebellum (84). This is partly because the vestibular system and cerebellum interactively control posture and ocular reflexes and produce multisensory models of sensory anticipation (13). Patients with cerebellar lesions present with language, visuospatial, executive function, and affective deficits, known as the Cerebellar Cognitive Affective Syndrome (85). It has been hypothesized that these deficits partly stem from altered inputs from the vestibular system which reduce patients’ ability to integrate sensory inputs, and in turn produces disorganized internal models and inaccurate timing predictions of real-world events (86).

Finally, Cochrane and et al. evidence a link between cognitive impairment in multiple sclerosis and detailed central sensory pathways that require the integration of vestibular information (87). Central vestibular measures including the VOR, subjective visual vertical, static balance, and functional gait tests were completed by patients with multiple sclerosis. The Brief International Cognitive Assessment for Multiple Sclerosis (information processing, verbal learning, and visuospatial memory) was also administered. The multiple sclerosis group performed worse across all central vestibular and cognitive measures relative to healthy control group. Moreover, performance on central vestibular measures was significantly correlated with cognitive performance (88).

In summary, the above findings provide evidence that disruption of sensory integration pathways involving the vestibular system leads to global deficits in cognition (51, 52). This contrasts with the profile of those with peripheral vestibular disorders which is characterized by more specific impairments to spatial memory and navigation.

## 4 Understanding the underlying mechanisms of effect

**Figure 1:**
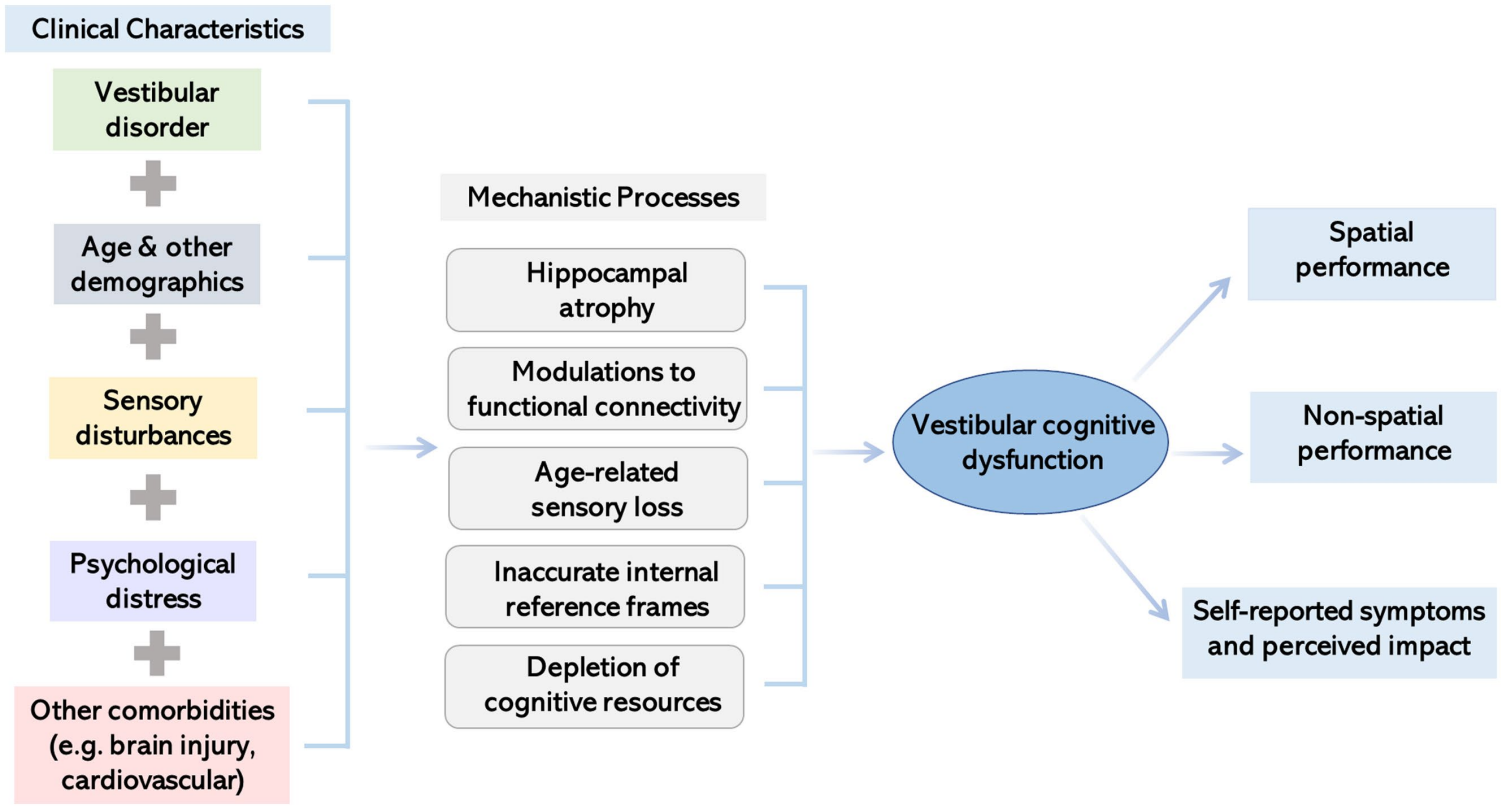
Illustration summarizing factors impacting the presentation of cognitive dysfunction in people with vestibular disorders and potential underlying mechanisms.

The previous section presents evidence of how vestibular dysfunction impairs cognition. This section will further explore the mechanistic bases of this psychological relationship. Since the development of vestibular cognitive dysfunction is likely multifactorial, several potential mechanisms are outlined below. Figure 1 summarizes current thinking around the mechanisms of effect.

### 4.1 Neuroanatomical

As described above, the vestibulo-thalamo-cortical projections are widely regarded as the primary means by which vestibular signals influence cognitive processing (50). In line with this, functional neuroimaging has revealed that caloric and galvanic vestibular stimulation activate multiple ‘cognitive’ regions including the anterior cingulate cortex, temporoparietal cortex, and prefrontal areas (14, 49). The insula integrates multisensory inputs with the internal state of the body and mind (interoception). The entire insula is activated by artificial stimulation of the vestibular system and is at the core of the vestibulo-cortical network (89). The parieto-insular component is strongly involved in the encoding of head and body movements to estimate head direction and produce egocentric representations of the self in space, while the posterior component is thought to integrate visual and vestibular cues to estimate head direction and distinguish between self-motion and object-motion, providing topographic and modality-specific interoceptive signals (39). By contrast, the anterior insula seems to play an important role in emotion regulation, it projects to the amygdala and autonomic nervous system which mediate fear and anxiety systems including avoidance behaviors (89). Together these functions indicate that the insula plays a significant role in integrating multisensory signals from external stimuli with internal interoceptive sensations to bring bodily consciousness and coordinate behavioral responses (90). Disturbances to these projections are thought to underlie several cognitive (hemi-spatial neglect, pusher syndrome, spatial disorientation (91)) and psychiatric disorders (dissociation, post-traumatic stress disorder (90)).

The spatial cognitive deficits and accompanying atrophy within the hippocampus and entorhinal cortex that appear in patients with vestibular failure have been attributed to the transmission of misinformation along the indirect pathways that project from vestibular brainstem to the hippocampus and parahippocampal area (13, 92). This underlines the importance of normal vestibular functioning and vestibular-hippocampal connections in spatial navigation in humans (46, 66, 93). The studies above indicate that vestibular-hippocampal connections are likely important for hippocampal processing of specific cognitive domains, i.e., spatial memory and navigation (13).

On a cautionary note, however, many of these attributions are founded on animal studies (using maze tasks) and on patients with bilateral or unilateral vestibular loss. Neuroimaging studies paired with different experimental paradigms conducted with other patient groups (including central or episodic peripheral conditions) would be valuable to explore whether the same neural pathways and cognitive domains are affected (56). This is important since the most commonly presenting conditions in the clinic tend to be BPPV, central vestibular vertigo, vestibular migraine, and Ménière’s disease (94).

Biochemical studies also indicate that vestibular inputs modulate several neurotransmitters implicated in cognition including glutamate (working memory, executive function) and dopamine (initiation, memory, goal-directed and reward-mediated behavior). Glutamate and dopamine receptors have also been identified in the saccule of the human vestibular system (95). Artificial stimulation of the vestibular system via externally applied thermal or galvanic current is also associated with the release of several relevant neurotransmitters including acetylcholine (attention, memory), Gamma-aminobutyric acid- GABA (inhibition, learning), and serotonin (attention, memory, cognitive flexibility, mood) (96).

### 4.2 Internal reference frames

Changes in vestibular signals alter the way in which other sensory cues are integrated and the formation of internal representations of allocentric and egocentric space. For example, distorted vestibular signals induce an erroneous perception of self-motion and body position in space, which in turn destabilizes the apprehension of space (97). This is illustrated by the errors shown by vestibular patients when judging their body position (i.e., egocentric-based transformation), mentally rotating objects, and navigating to a target from memory (8). Caloric and galvanic vestibular stimulation can also remediate symptoms of hemi-spatial neglect, a condition characterized by disturbances to multimodal spatial representations (98, 99). Changes in internal spatial representations might be foundational to vestibular cognitive dysfunction, contributing to dysfunction in other non-spatial domains (e.g., arithmetic, object memory, self-identification) (47, 52). To explore this idea, further investigations could utilize latent measures to elucidate how performance on a cognitive task is affected by multiple cognitive processes and whether deficits in one cognitive domain contributes to dysfunction in other domains.

### 4.3 Attentional resources

Attentional capacity demands are also thought to provide a mechanism for cognitive dysfunction in vestibular disorders. This model places less emphasis on specific cognitive domains, instead focusing on the cognitive effort required for balance maintenance. Dual-task studies have demonstrated competition for cognitive resources, producing interference effects when participants are required to perform a postural exercise at the same time as a cognitive task (100). Results have been variable, partly because of the different study designs applied (101) but also because of varying degrees of vestibular loss and anxiety (fear of falling) across participant samples which could impact whether cognitive or postural performance is prioritized during the task (8). Lacroix et al. (62) suggest that the ‘cognitive cost’ of coping relates to the degree of vestibular compensation which might help explain some variability. This hypothesis predicts that patients with higher levels of dizziness handicap and less physiological compensation will show more preserved cognitive function, while those with reduced levels of dizziness handicap and greater compensation will show more impaired cognitive function. Most dual-task paradigms are typically administered under ideal conditions designed to minimize cognitive distractions and limit head movements, meaning they are unlikely to capture patient-reported real-world deficits in multitasking where a ‘cognitive cost’ is experienced (e.g., remembering items at the supermarket, walking the route back to the station after dark). Future research should include cognitively demanding tasks which challenge cognitive effort to resemble daily activities.

### 4.4 Sensory disturbances

Vestibular disorders can also impact cognitive functioning indirectly. Subtle decrements in oculomotor and postural control could affect cognitive performance if patients are unable to see clearly (e.g., reading, viewing quick moving stimuli) or move properly (e.g., walking to a point of interest, turning head during conversations) (47, 52). Somatosensory information can be amplified and distorted, eliciting subjective dizziness that is exacerbated by motion and with visual stimulation. Secondary effects including fatigue, avoidance, and cognitive problems can also develop (102). Patients may compensate over time, especially if they have received treatment for their symptoms (e.g., vestibular rehabilitation, medication, psychological therapies (103)). Longitudinal designs where participants are tested across multiple time points will therefore be important to gain reliable estimates of vestibular cognitive dysfunction and capture compensation within chronic conditions (62).

The vestibular and auditory systems are also closely intertwined; both are located in the inner ear and share the vestibulocochlear neural pathways with the saccule and utricle also responding to sound (104). Vestibular disorders and hearing loss often occur together (e.g., Ménière’s disease, enlarged vestibular aqueduct syndrome, age-related presbyataxia and presbyacusis) and reflect the labyrinthine and cochlea effects of the underlying pathology. It is also noteworthy that auditory stimulation affects hippocampal place cell function (105, 106), and that hearing loss is the largest modifiable risk factor of dementia (107). Hearing loss could therefore be contributing to cognitive dysfunction in vestibular disorders where the two co-exist (108). Smith (48) reviewed 19 studies of vestibular cognitive dysfunction which controlled for hearing loss, finding at least some cognitive effects (mostly spatial) were likely to be driven by vestibular dysfunction not hearing loss. Recently, Daneels et al. (109) also evidenced an association between vestibular function and dual-task performance involving cognitive and motor components, while the association held in patients with isolated bilateral vestibulopathy, it was stronger among those with concomitant hearing loss. Moving forward, both hearing and vestibular status should be objectively evaluated (not self-reported) within studies to help establish the relative contribution of each to any observed cognitive dysfunction.

Tinnitus is the perception of sound without any external sound source and is associated with hearing loss. Tinnitus is common affecting approximately 15% of the population, and prevalence increases with age (110, 111). Those with a recently diagnosed vestibular or ontological disorder are also at higher risk of being diagnosed with tinnitus (112), particularly Ménière’s disease and vestibular migraine (estimated prevalence of 49 and 39.6%, respectively (113),), for which tinnitus may be the first symptom of the vestibular disorder (114). Shared neural fiber connections between the cochlear and vestibular system, and changes in the hydrodynamics of inner ear fluids are thought to underlie this association, at least in Ménière’s disease (115). Stress can also trigger tinnitus, and psychological distress (depression and anxiety) frequently occurs in response to the impact of tinnitus (111).

Multiple auditory and non-auditory brain areas are activated by tinnitus percepts. These include the auditory cortex, limbic system (amygdala, hippocampus, hypothalamus, and cingulate cortex), prefrontal cortex, and insula (116, 117). These sub-networks are thought to relate to emotional and cognitive processes including persisting attention toward tinnitus sounds, multisensory integration, forming auditory memory of tinnitus percepts, and emotional distress of coping with bothersome and intrusive symptoms (118).

Tinnitus patients frequently self-report cognitive difficulties, mainly affecting focal attention, concentration, and short-term memory. However, objective evidence of working memory and attentional (alerting sustained, selective) impairment is equivocal (119). Some domains of executive function appear to be consistently affected, particularly the executive control of attention (120). More research is required to understand the interactive relationship between tinnitus and cognition, establishing which cognitive domains are affected and why. Preliminary findings indicate that poorer signal in noise recognition might underlie cognitive difficulties when processing speech in noise (121). Research should also delineate the additional impact of co-existing psychological distress and hearing loss, as well as aging since these factors can impede cognition independently (120, 122). Future studies could also compare the cognitive profiles of vestibular patients who present with tinnitus related to those who do not (123, 124).

### 4.5 Aging

Aside from vestibular injury and disease, cognitive dysfunction can also result from natural age-related losses in vestibular function (i.e., decreases in hair cells of the peripheral organs, fewer neurons in vestibular nerve and nucleus) (125). Age-related vestibular declines are associated with reduced visuospatial ability in healthy older adults (126). Vestibular dysfunction is also more prevalent among patients with mild cognitive impairment (MCI) and Alzheimer’s disease relative to age-matched controls (127). Moreover, the clinical presentation of this sub-group is characterized by spatial cognitive deficits which significantly impact health and morbidity (including wandering, difficulty driving, disorientation, increased fall risk) (128, 129). Declines in vestibular inputs are thought to impact regions within the vestibular cortical network (e.g., the hippocampus, posterior cingulate, and parietal–temporal cortex) (130), with hippocampal atrophy thought to be a key neuropathalogical mechanism (131). Importantly, hearing loss was not previously controlled (127–129) but is likely to be a contributing factor to Alzheimer’s disease (106). A longitudinal study is currently underway to assess the effect of hearing and vestibular loss on cognition in people with MCI and Alzheimer’s disease (132).

### 4.6 Social deprivation

The vestibular system adds automatism to activities via the VOR, vestibulo-collic reflex, and vestibulo-spinal reflex. Disorders affecting vestibular reflexes can blur vision during head movements, impair postural control, slow walking, and reduce cognitive function. Since these reflexes cannot be replaced by other sensory systems, patients may need to slow, modify, or avoid activities of daily living (133). Mixed method approaches indicate this involves restricting participation in meaningful activities (e.g., gardening, household duties, socializing, work) which reduces psychological wellbeing and health-related quality of life (134–136). Vestibular impairment also increases risk of falls and fear of falling, which can further reduce activity and contribute to more disability (133).

Restricting activities in this way may increase the risk of social deprivation. Social activity is a modifiable risk factor for dementia (107) and is thought to influence cognition through social stimulation and environmental enrichment since these increase cognitive reserve and brain plasticity (137). Individuals with high social deprivation are also more likely to experience chronic stress (e.g., work, financial) which can induce harmful neuroinflammation and neurodegeneration (138). Moreover, social deprivation is associated with depression and loneliness which can further exacerbate cognitive impairment (137).

The COVID pandemic provided a unique illustration of the effects of social distancing and prolonged social deprivation. People with vestibular disorders self-reported their concentration and memory had worsened during the pandemic while their daily activities were restricted (139). Moreover, patients with other neurological conditions (dementia and Parkinson’s) showed rapid objective cognitive decline during the lockdown period in comparison to the pre-lockdown period (140, 141). Further research is needed to explore the cognitive consequences of social deprivation among people with vestibular disorders and devise effective interventions to promote participation.

### 4.7 Psychological factors

Vestibular disorders frequently co-present with psychological distress (4). Anxiety, depression, and phobic disorders appear to be particularly prevalent. Patients with episodic excitatory vestibular conditions (e.g., vestibular migraine, Ménière’s disease, vestibular paroxysmia) (37, 142–144) appear most susceptible as well as those with a pre-existing psychiatric condition (145). Psychological distress can instigate and aggravate dizziness and prolong the impact of vestibular disorders, with complex vicious circles of interaction at play (dizziness symptoms worsen psychological wellbeing, which in turn worsens perceived severity of dizziness symptoms) (146, 147). This bi-directional association must be considered, since psychiatric conditions (in those with no vestibular disorder) are known to impact cognitive function (148), implying that vestibular cognitive dysfunction could be both psychological and vestibular in origin (46, 52, 65). Of particular relevance here, depression can impair working memory and attention (149, 150), while spatial working memory and attentional control are affected in anxiety disorders (151).

Psychological distress can emerge as a reaction to the impact of experiencing and living with a vestibular disorder and the personal losses experienced (142). Anxiety, panic, and catastrophic thoughts often accompany vestibular symptoms (e.g., vertigo and nausea) serving as reinforcing stimuli for the conditioned avoidance of situations that evoke discomfort (e.g., busy supermarkets, riding on escalators), anxiety can also generalize if individuals become hyper-vigilant (152). Attentional Control Theory (153) suggests that these worrisome thoughts can indirectly impair cognitive functioning by increasing attention toward threat-related stimuli and decreasing the availability of resources for maintaining attentional control (inhibition and shifting) and goal-directed attention, thus impeding upon memory and other related cognitive processes.

Psychological distress could also be considered a brain disorder in the same way as the cognitive processes discussed above (37). Vestibular hippocampal interactions could induce psychiatric symptoms since the hippocampus is implicated in both spatial and emotional processing (46). Neurotransmitter changes in the serotonergic and dopaminergic systems are implicated in several vestibular syndromes and anxiety and depression (50, 148). Moreover, there is close structural overlap between the networks relevant for vestibular function and fear conditioning, including the insula, amygdala, secondary somatosensory cortex, dorsal anterior cingulate cortex, and thalamic areas (89). Personality traits (neuroticism, introversion) and psychological distress (anxiety, depression) can also modulate functional connectivity in brain regions involved in multisensory processing (vestibular and visual cortices), spatial cognition (hippocampus, cerebellum) and emotion regulation (hippocampus, frontal cortex) (154). These brain changes could reflect maladaptive coping including an over reliance on visual information, deficits in spatial perception, and heightened anxiety toward triggering symptoms. Multiple neuropsychiatric symptoms (including psychiatric, cognitive and fatigue disturbances) could therefore arise due to changes to the vestibular cortical network, rather than an exclusive neural link between cognition and vestibular dysfunction (4).

Finally, there is a smaller distinct group of patients with psychogenic (also termed somatoform and functional) dizziness where the underlying pathology is inconsistent with a vestibular origin. These patients show no structural abnormality of the inner ear or central connections, more likely reflecting psychosomatic disturbance (155). There is limited data on the cognitive profile of this group, however, preliminary evidence indicates this may differ to that of people with vestibular disorders, since verbal memory and attention switching are primarily affected (156), while short- and long-term memory are relatively unaffected (157). Dizziness Handicap Inventory (DHI) scores also differed significantly between neuro-otology patients with structural, functional, and psychiatric disorders (158). Further research with carefully characterized samples (no organic medically recognized vestibular disorder or other neurological condition) could provide useful insights into the differential effects of vestibular and psychiatric disturbances on cognitive function.

## 5 Assessing vestibular cognitive dysfunction

Table 1 summarizes the most commonly used assessments of cognitive function within vestibular cognitive research. It highlights a range of experimental tasks and neuropsychological assessments, self-report questionnaires, screening tests and neuropsychological batteries that cover several cognitive domains.

**Table 1:** Review of commonly used cognitive assessments and recommendations for implementation.

Assessment approach	Examples	Advantages	Disadvantages	Recommendations
Experimental tasks	Virtual Morris Water Maze Triangle Completion Task Path Trajectory Dual Task Reaction Time (simple, choice, inhibitory) Mental Rotation Arithmetic/Counting Line Matching Sentence Completion Stroop	Computerized testing captures precise data (reaction time, path length) Carefully controlled designs for hypothesis testing Dynamic testing to active vestibular processing	Lack ecological validity Normative data unavailable Lengthy and require specialized equipment	Use in research settings to develop theories that inform clinical practice Sharing materials on open science platforms to aid replication and validation Utilize well-matched control groups Explore across a range of vestibular disorders (beyond bilateral/unilateral vestibular loss) Combine with fMRI to explore structure/function relationships
Cognitive screening tests	Montreal Cognitive Assessment (MoCA) Mini Mental State Examination (MMSE)	Normative data available Quick and practical to complete Widely recognized	Lack specificity for vestibular cognitive domains Insensitive to mild/moderate cognitive impairment	Apply in clinical settings for cognitive screening with older adults or suspected severe cognitive impairment Share scores with multidisciplinary team to inform care provision
Neuropsychological assessments	*Memory* Wechsler Memory Scale, Doors Test. Digit Span, Brooks Spatial and Non-Spatial Memory, Corsi Block, Paired Associates Learning, Benton Visual Retention Test *Executive function* Trail Making, Digit Symbol Substitution *Language* California Verbal Learning Test, Category and Letter Fluency *Visuospatial* Money Road Map Test, Rey-Osterrieth Complex Figure Task	Normative data available Select to target specific cognitive domains Customize to an individual or hypothesis	Training often required to administer and interpret Copyrighted (license fees, not widely available) Can be lengthy to administer	Form a consensus on which assessments are most relevant to vestibular cognition Produce consensus suite of assessments to facilitate comparisons between research studies and guide clinical practice Gather more data with well-defined vestibular groups, and matched controls Digitized assessments could be completed remotely to reduce demands on clinical teams and improve patient comfort
Neuropsychological assessment batteries	Wechsler Adult Intelligence Scale Repeatable Battery for the Neuropsychological Status	Normative data available Widely recognized Provide comprehensive evaluation of cognitive profile	Training required to administer and interpret Copyrighted (license fees, not widely available) Some assessments within the battery lack specificity for vestibular cognitive domains Lengthy administration times	Utilize for more comprehensive clinical (geriatric, neuropsychology, pediatric) and research evaluations To inform hypotheses and intervention development Explore construct validity of experimental assessments
Self-report	Dizziness Handicap Inventory (DHI) Vertigo Symptom Scale (VSS) Neuropsychological Vertigo Inventory (NVI) Cognitive Failures Questionnaire Everyday Memory Questionnaire (EMQ)	Express perceived impact according to individual High intrasubject reliability Quick to complete Capture fluctuating symptoms across wider time window	Not a direct measure of cognitive ability Psychological distress influences self-ratings Often uncorrelated with objective cognitive tasks and vestibular function testing Assessments with 1 or 2 items on cognitive function provide limited perspective	Use broad measures (e.g., DHI) as a discussion and triage tool within clinical settings Use focused measures to make inferences about cognition or treatment outcomes (e.g., NVI, EMQ) Combine with objective cognitive assessments and affective questionnaire for more complete picture Consider questionnaires with composite scores for affective and non-affective domains

In the United Kingdom (United Kingdom), primary care physicians, neurologists, neuro-otologists, audiologists, or vestibular physiotherapists are usually the first clinical point of contact for patients with vestibular disorders. In such busy clinics, it is difficult to incorporate either a psychiatric or cognitive assessment. Hence cognitive assessment and monitoring are not routinely offered to people with vestibular disorders within the clinical care setting (159). This practice is not only borne by pressing time constraints, but also the lack of consensus guidelines surrounding appropriate assessment tools (160). There is usually no joint working across the disciplines of psychiatry, psychology, and neuro-otology when it comes to managing the care of patients with vestibular disorders.

Where provided, cognitive assessment in the United Kingdom typically involves completing a questionnaire with only one or two items capturing cognitive functioning (160) such as the DHI (161) or Vertigo Symptom Scale [VSS, (162)]. This provides a starting point for discussions about cognitive problems, however there is a lack of consensus around the assessments that should be administered to those patients who require further screening and, in some cases, comprehensive evaluation.

To improve practice, the field would benefit from defined care pathways involving clinicians across specialties. In addition, a Delphi consensus study conducted with interdisciplinary researchers and clinicians, and people with vestibular disorders would help to assess the acceptability of different tools and develop a recommended set of assessments that can be routinely applied to screen for cognitive dysfunction and capture outcomes following treatment within clinical settings or randomized controlled trials. No core outcome sets of relevance to vestibular cognition are available on the Core Outcome Measures in Effectiveness Trials (COMET) database (163). Vestibular cognitive dysfunction typically has a subtle presentation, affecting specific cognitive domains, at a mild to moderate level (52). Generic measures may therefore fail to identify relevant aspects of cognitive functioning impacted in vestibular disorders and lack the specificity to form vestibular cognitive profiles. For example, the MoCA is commonly applied but includes domains not commonly affected in vestibular disorders (e.g., orientation to time and date), while navigation and multitasking are absent. Therefore, the design and selection of assessments capable of assessing cognitive domains most impacted by vestibular disorders is important (164).

Assessment selection should also consider the delivery format to account for sensory difficulties experienced by people with vestibular disorders (e.g., hearing loss and tinnitus in Ménière’s disease, and visual dominance in vestibular migraine and Persistent Postural Perceptual Dizziness (PPPD)), which are not cognitive in nature (52). Self-report measures are likely to be important in understanding the perceived impact of vestibular cognitive dysfunction, particularly until more specific and naturalistic paradigms are developed (165). The Neuropsychological Vertigo Inventory targets vestibular cognitive domains (affective state, temporal memory, spatial memory, visual spatial cognition) across 22 items (164, 166). Developing a briefer version of this questionnaire may prove more practical for in-clinic monitoring. Anxiety, depression, and stress are also prevalent among people with vestibular disorders and correlate with self-reported cognitive dysfunction (167). An assessment of psychological distress is therefore likely to be important to form an accurate clinical picture. Several questionnaires of anxiety and depression are freely available and have been validated with vestibular cohorts (e.g., Patient Health Questionnaire, Generalized Anxiety Disorder Assessment (168)). Alternatively, some vestibular self-report measures include separate subscales for affective components of vestibular dysfunction (e.g., DHI, VSS) which lend themselves to clinical contexts. A newly validated Balance Vigilance Questionnaire (169) and Updated Perceived Control over Falling Scale (170) are also freely available to identify maladaptive hypervigilant behaviors that may lead to avoidant behaviors that limit clinical engagement.

As mentioned, time constraints within the clinic present a barrier to routine cognitive monitoring of people with vestibular disorders (160). Therefore, there is a need to develop brief screening tools which can be administered routinely, as well as comprehensive neuropsychological batteries for further insights. Computerized screening tools may be a viable alternative to conventional paper-and-pencil assessments, requiring less input from clinical teams and allowing patients to complete assessments within more comfortable environments including their homes. The electronic data generated could be integrated in medical records to implement cognitive monitoring as part of standard care (171) and combined across sites into large datasets, like the Ménière’s Disease Registry (172), to advance understanding of the prevalence and time course of cognitive dysfunction across different vestibular conditions. Affordable commercial digital tools are however necessary for this to be sustainable for health services.

Finally, we highlight the need to make better use of standardized assessments and reporting (i.e., how assessments are administered, scored, and analyzed) to enable interpretation and facilitate comparisons across studies. Administering standardized assessments to large, well-characterized samples matched across important demographic characteristics (including age, gender) and utilizing regression models to adjust for potential confounding factors (including hearing loss, tinnitus, and psychological distress) could also enable the classification of cognitive dysfunction into further categories, such as ones based on severity (i.e., mild, moderate, and severe), vestibular etiology (e.g., peripheral, or central), or cognitive phenotypes (e.g., spatial, or attentional). These categories could assist with triaging people with vestibular disorders to receive further testing and/or cognitive rehabilitation earlier in their care pathway and help inform the treatment strategy.

## 6 Prevention and treatment of cognitive problems in vestibular disorders

Improved understanding of the profile of vestibular cognitive dysfunction and the mechanisms underlying this are important for developing targeted treatments. Effective pharmacological treatments require knowledge of the neurotransmitters implicated in vestibular cognitive dysfunction, while cognitive rehabilitation interventions necessitate an understanding of affected cognitive domains and behavior change processes to be targeted (173). Similar principles apply for vestibular rehabilitation interventions (exercises designed to promote vestibular compensation), whereby clinicians must first identify the nature of vestibular disturbance to plan a program around these physiological mechanisms. Given the uncertainty in the pathogenesis of vestibular cognitive dysfunction, at present no clear mechanism of action has been established for pharmacological or non-pharmacological interventions targeting cognition. Consequently, there are no specific recommendations for how to assess and treat cognitive problems. The provision of cognitive support is therefore open to interpretation by individual healthcare professionals, contributing to variation in clinical practice (160).

### 6.1 Current treatment approaches

Treatment typically focuses on treating the underlying vestibular disorder with the assumption that, if cognitive problems are directly related to vestibular disorder, then treating the root cause may also resolve patients’ cognitive deficits (4). Treatment for vestibular disorders varies according to the disorder and stage of recovery but common approaches include vestibular rehabilitation, pharmacological therapies, dietary and lifestyle modifications, and surgery (9). Therapies designed to target psychological distress (e.g., anti-depressants, relaxation techniques) may also be prescribed with the assumption that cognitive deficits are a secondary consequence of psychological distress and will therefore resolve once affective symptoms are controlled (4).

Currently there are no evidence-based psychopharmacological interventions specific for the neuropsychiatric complications of vestibular disorder, including cognitive dysfunction. Treatment is largely based on clinician-led experience. Clinicians must select medications carefully to treat the different aspects of vestibular disorders. For example, antiepileptics medications (topiramate, gabapentin, and pregabalin) and tricyclic anti-depressants (nortriptyline and amitriptyline) are frequently required to treat vestibular migraine and can potentially affect cognitive function and cause fatigue which could exacerbate cognitive dysfunction (174).

Typically, only those with severe vestibular-related psychopathy are seen in neuropsychiatric clinics, usually after years of distress and lack of symptom relief in primary care, even after attending specialist neuro-otology clinics (4). The subsequent management of psychiatric symptoms proceeds as for the management of these problems in any psychiatric patient. There is limited evidence of the long-term cognitive benefits or harm due to psychiatric interventions in vestibular disorders. However, the clinical consensus favors early intervention for prognostic reasons (147, 175, 176).

Targeted vestibular rehabilitation exercises designed to strengthen substitution, habituation, and adaptation mechanisms through targeted exercises can improve cognitive performance among patients with bilateral and unilateral vestibular loss (spatial working memory (177)) and persistent dizziness (executive function (178)). Cognitive behavioral therapy (CBT) techniques designed to help individuals challenge dizziness-related beliefs by recognizing how their thoughts (cognition) influence emotions and behaviors has also shown beneficial effects on vestibular symptoms (e.g., tinnitus (179)) and vestibular sub-groups (e.g., PPPD, functional dizziness) (94, 180). Moreover, combined approaches where CBT and vestibular rehabilitation are delivered concurrently (181, 182) appear to reduce dizziness handicap, psychological distress, and avoidance. Future studies could include a cognitive assessment as a secondary outcome measure to explore whether CBT also attenuates the severity and/or perceived impact of cognitive problems.

Cognitive training interventions designed to enhance cognitive skills (memory, attention, executive function) through behavioral interventions have improved balance in older adults (183, 184), including those with MCI and Alzheimer’s disease (184). Higher doses of cognitive training (i.e., more sessions) also appear to mitigate physical functioning declines in older adults (185) with simultaneous and sequential interventions that combine cognitive and physical training appearing more efficacious than single-domain training (186). Looking ahead, a study to explore a combined intervention where cognitive rehabilitation techniques (training to restore cognitive functions or compensatory strategies to adapt to cognitive deficits) and vestibular rehabilitation exercises are delivered concurrently therefore seems worthwhile for vestibular patients. Further randomized controlled studies with active (an intervention not designed to target cognitive skills of interest) and passive (no-intervention) control conditions might also be helpful to elucidate which components of the intervention are driving the treatment effect (e.g., contact with a health professional, cognitive training, changes in everyday behaviors) (187). Details of the intervention protocols and theoretical models are often lacking limiting replication and translation into clinical settings (188). Checklist tools such as the Template for Intervention Description and Replication could help researchers provide more complete descriptions of their interventions to advance the field (189).

### 6.2 Future directions

Evidence for cognitive interventions in vestibular disorders is scant. Ellis et al. (190) outline a cognitive training model for bilateral vestibular loss involving mental imagery training where head and body movements are simulated to improve patients’ knowledge of head movement dynamics. Cognitive training exercises could be designed to harness intact higher-level cognitive functions (mental imagery, executive functions, attention) to improve predictions of self-motion perception, by increasing the weighting of prior knowledge and inhibiting the weighting of uninformative sensory input from the vestibular end organs, by reallocating attentional resources toward internal estimates. This model now needs to be tested to evaluate its impact on patients with bilateral vestibular loss, and if effective, translated to clinical settings.

Given that vestibular decline is associated with age-related cognitive impairment, and cognitive impairment influences balance and gait (191), vestibular rehabilitation exercises could also form part of a preventative strategy to protect cognitive reserves and promote healthy aging (48, 126). As intimated above, vestibular rehabilitation could be a potentially beneficial therapy for dementia patients. To this end, we note that a pilot randomized controlled study is currently underway to explore the impact of vestibular rehabilitation on falls and cognitive outcomes (clock drawing test, Card Rotations test, Money Road Map test, triangle completion task) among Alzheimer’s disease patients (192).

On a similar note, a growing literature indicates that artificial stimulation of the vestibular organs via galvanic or caloric waveforms can remediate a range of cognitive and affective difficulties (193, 194). This literature is mostly focused on individuals with a central nervous condition rather than a vestibular disorder, and few have investigated whether cognitive improvement following vestibular stimulation correlates with improvements in balance. Those stimulation studies that have recruited vestibular patients tended to focus on balance rather than cognitive outcomes and together have produced mixed results (195, 196). That said, if there is a subset of vestibular deficits (perhaps most promisingly in bilateral vestibulopathy (197)) that respond favorably to vestibular stimulation, then one might expect to see allied cognitive improvements if fewer attentional processes now need to be diverted to control balance. More generally, given the known inter-dependency between cognitive and emotional function, it may be that the improvements in mood and well-being induced by galvanic or caloric artificial stimulation mitigate some aspects of cognitive dysfunction (98, 198), regardless of whether the underlying vestibular deficit has also responded.

Despite the potential merit of cognitive interventions, there is an international shortage of professionals (psychologists, neuropsychologists, occupational therapists) able to deliver them (199). In the absence of such specialist referral, it is especially important for vestibular healthcare professionals to be aware of the cognitive consequences of vestibular disorders so that they can discuss these with their patients, validate their concerns, and help normalize their experiences, therefore minimizing some of the distress experienced (200). Some vestibular healthcare professionals could play a greater role in detecting cognitive dysfunction earlier, include this within their clinical decisions, and monitor cognitive outcomes in response to their treatment of the underlying vestibular disorder. They could also signpost patients toward appropriate psychoeducation resources and proactively review these with them.

Vestibular healthcare professionals could also consider integrating some basic cognitive rehabilitation techniques (e.g., compensatory strategies, pacing, encouraging the use of external aids) within their practice. Cognitive rehabilitation appears most effective when tailored to the individual to account for their specific needs (201), and when delivered with input from a healthcare professional (202). Integrating cognitive rehabilitation with vestibular rehabilitation sessions delivered by a physiotherapist may offer a practical solution for clinical implementation and will likely help to maximize therapeutic gains, since patients with cognitive impairment tend to have poorer outcomes on standard vestibular rehabilitation programs (203). Our experience tells us that vestibular healthcare professionals are already doing some of this (e.g., adapting the care they deliver to accommodate for cognitive impairments, assisting patients to comply with treatment regime), but further guidance from psychology professionals and training opportunities would help to develop their practice further (160).

Self-management interventions are also likely to form an important therapy component (204). Self-management interventions for cognition include booklet-based techniques, homework tasks, cognitive rehabilitation computer programs, and ‘brain training’ games. These have shown some efficacy in people with MCI and dementia (183) and other neurological conditions with overlapping symptoms including multiple sclerosis (205).

## 7 Conclusion

This narrative review summarizes key literature and recent updates related to vestibular cognitive dysfunction. Our aim has not been to provide an exhaustive review, but rather to highlight important points that may be of practical value to healthcare professionals treating patients and to researchers considering further studies in this field.

We highlight how the diffuse and widespread cortical and subcortical projections of the vestibulo-cortical network provide a neuroanatomical substrate for the cognitive and psychiatric sequelae frequently seen in vestibular disorders. These complex, multimodal, reciprocal, and non-reciprocal pathways with brain areas implicated in cognitive and psychological function are reflected in the multifaceted neuropsychiatric symptoms of people with vestibular disorders. They also suggest a substantial degree of redundancy within the central vestibular pathways which is reflected in the remarkable ability, generally, of patients to compensate for peripheral vestibular deficits and, with appropriate treatment, to recover or cope with most vestibular disorders.

Vestibular signals appear to make a specific contribution to spatial cognition (memory, perception, navigation), likely via their modulation of hippocampal and parietal spatio-topic maps. Vestibular signals also impact upon other non-spatial cognitive domains (object perception, arithmetic), likely through their contribution to allied internal reference frames. Competition for cognitive resources and compensatory mechanisms are also thought to contribute to the deficits observed in non-spatial cognitive domains.

We also reviewed other concurrent factors which can influence cognitive performance. Hearing loss and tinnitus are associated with cognitive impairment and commonly present in vestibular disorders. Both are more common with increasing age which exerts a separate effect on cognitive function. Psychological distress is also prevalent in vestibular disorders and can exacerbate vestibular symptoms. A further layer of complexity is added by the fact that psychiatric conditions are also associated with cognitive deficits. At the anatomical level, many of the brain areas within the vestibulo-cortical network overlap with emotion regulation and are implicated in psychiatric disorders. The interdependence of confounding clinical characteristics has likely been a major factor contributing to the difficulty in establishing clear, well-defined causal links between vestibular dysfunction and cognitive deficits. To address this complexity, research could benefit from recruiting carefully characterized samples with various vestibular pathologies, using standardized psychological assessments, and applying longitudinal designs. Those efforts will likely increase the probability of better understanding how cognitive profiles vary across individuals and vestibular etiologies, the duration of vestibular cognitive dysfunction, and how we can best treat them. Healthcare professionals should explore these concurrent factors within their workup to form an accurate cognitive profile and develop an effective treatment strategy.

In closing, given their inextricable links, one wonders if we will ever be able to completely delineate vestibular–cognitive–psychological interactions. But as in any area of medicine, greater insight may not only prompt more targeted treatment, but may also reassure patients. Consequently, for the many patients presenting with a complex combination of vestibular, cognitive, and psychological disturbance, we suggest that psychoeducation to briefly explain and normalize this triad of symptoms is likely to be the first and most important step in their treatment.

## Author contributions

LS and SS contributed to the conceptualization, design, and investigation of the review. LS and SS wrote the first draft of the manuscript. DW and MB reviewed and edited the manuscript. All authors contributed to the article and approved the submitted version.
